# Risk Factors and Referral Rates for Urinary Tract Infection in Pregnant Mothers of Southwest Guatemala

**Published:** 2021-10-09

**Authors:** Michael Miller, Molly Lamb, Claudia Rivera, Saskia Bunge Montes, Andrea Jimenez-Zambrano, Antonio Bolanos, Edwin Asturias, Stephen Berman, Margo S Harrison

**Affiliations:** 1 University of Colorado, School of Public Health, Center for Global Health, Colorado, USA; 2 FUNSALUD, Guatemala, Mexico; 3 Department of Obstetrics and Gynecology, University of Colorado, Colorado, USA

**Keywords:** Urinary Tract Infection, Pregnancy, Latin America, Guatemala

Urinary tract infections (UTIs) affect one in four women in low- and middle- income countries and are associated with adverse maternal and neonatal outcomes [1, 2]. In Latin America, the prevalence of UTI is estimated to be 23–31%, yet data in this region is sparse [3]. The objective of this analysis was to understand the prevalence, referral rates, and risk factors associated with UTIs in the Southwest Trifinio region of Guatemala.

This study is a secondary analysis of a prospective cohort enrolled in the Madres Sanas community prenatal nursing program in Southwest Guatemala from 2018 to 2020. Study approval was obtained from the Colorado Multiple Institutional Review Board. Women enrolled in the Madres Sanas program who provided at least one dipstick urine sample and had a documented birth were included in the cohort. The study’s primary outcome was presence or absence of UTI as indicated by dipstick (leukocyte esterase count > 15, protein ≥ 30 mg/dL, or a positive result for nitrites) at any point during four antenatal visits. SAS software University Edition version 9.4 was used for analysis (SAS Institute Inc., Cary, North Carolina).

From 2018 to 2020, 344 pregnant women were included in this cohort. On average, the population was 24 years old and had 2 prior pregnancies. The majority of the population received some formal (primary or higher) education, were married, had a weekly income ≤ $5 USD, and were not employed by the local agribusiness. Prevalence of UTI among the cohort was 12.8% with an incidence of 60.9 per 1,000 individuals. Rate of referral for positive UTI was 32.1%, and UTI accounted for the greatest number of referrals compared to all other reasons for referral. Rate of repeat positive dipstick was 15.9%, though it was unknown whether this was the result of reinfection or inadequate treatment. No significant differences in bivariate analyses were observed between women with and without diagnosis of antepartum UTI (data not shown). Pregnant mothers with no formal education had a non-significant increased risk of UTI (aRR 1.8; 95% CI: 0.94, 3.32), and mothers working for the local agribusiness had an increased but non-significant risk of UTI (aRR: 2.4; 95% CI: 0.89, 6.18) in a generalized linear regression model (Table 1).

Based on our results, we conclude there is a need for prospective research on UTI in this population with confirmatory culture, and observation of treatment practices and pregnancy outcomes associated with UTI diagnosis.

## Figures and Tables

**Table 1:** Crude and adjusted risk ratios for education and employment at Banasa and risk of urinary tract infection.

	RR	95 CI	p-value	aRR	95 CI	p-value
**Education Level**						
No formal education	1.8	(0.9, 3.4)	0.07	1.8	(0.9, 3.3)	0.08
Some Education	-	-	-	-	-	-
**Employed**						
Yes	2.4	(0.9, 6.5)	0.08	2.3	(0.9, 6.2)	0.09
No	-	-	-	-	-	-
